# Anticipatory Manoeuvres in Bird Flight

**DOI:** 10.1038/srep27591

**Published:** 2016-06-08

**Authors:** Hong D. Vo, Ingo Schiffner, Mandyam V. Srinivasan

**Affiliations:** 1 Queensland Brain Institute, University of Queensland, St Lucia, QLD 4072, Australia; 2 School of Information Technology and Electrical Engineering, University of Queensland, St Lucia, QLD 4072, Australia; 3 ARC Centre of Excellence in Vision Science, University of Queensland, St Lucia, QLD 4072, Australia

## Abstract

It is essential for birds to be agile and aware of their immediate environment, especially when flying through dense foliage. To investigate the type of visual signals and strategies used by birds while negotiating cluttered environments, we presented budgerigars with vertically oriented apertures of different widths. We find that, when flying through narrow apertures, birds execute their maneuvers in an anticipatory fashion, with wing closures, if necessary, occurring well in advance of the aperture. When passing through an aperture that is narrower than the wingspan, the birds close their wings at a specific, constant distance before the aperture, which is independent of aperture width. In these cases, the birds also fly significantly higher, possibly pre-compensating for the drop in altitude. The speed of approach is largely constant, and independent of the width of the aperture. The constancy of the approach speed suggests a simple means by which optic flow can be used to gauge the distance and width of the aperture, and guide wing closure.

While considerable effort has been devoted to understanding how birds navigate over long distances, we know relatively little about how they guide their flight through cluttered environments. Flight through dense foliage, for example, requires the ability to avoid collisions with obstacles, to choose quickly between alternative routes, and to ensure safe flight through narrow passages^1^,^2^,^3^,^4^. There is evidence to suggest that budgerigars steer through the middle of a corridor by balancing the magnitude of optic flow (the speed of image motion) experienced by the two eyes^5^. Furthermore, budgerigars seem to be exquisitely ‘body aware’: when preparing to fly through a narrow aperture, they close their wings only when the width of the aperture is smaller than their wingspan, demonstrating an ability to gauge aperture width in relation to wingspan with a precision of about 1 cm^2^. Similarly, pigeons, when flying through narrow apertures, adopt two different postures, depending upon the width of the aperture. When the aperture is relatively wide, they pass through with the wings held in the vertical position; when it is narrow, they fold their wings right back^3^. The execution of such intricate manoeuvres requires selection of the appropriate motor action, as well as execution of these actions at the correct point in time or space. Gannets plunging into the ocean to catch fish fold their wings back in preparation for entry into the water at a fixed time prior to contacting the water surface^6^. Hawks landing on a perch extend their talons at a fixed time prior to touchdown^7^.

How do birds orchestrate their flights when they fly through a narrow aperture? Here we film budgerigars as they take off from a perch, fly through an aperture of variable width, and land on a perch on the other side. The video data is analysed to measure the speed at which the bird approaches the aperture, and to determine the position and time at which it closes its wings. The analysis should enable us to understand how birds estimate the width of the aperture in relation to their wingspan and to determine whether wing closure is necessary or not, and if so, when (or where) it should occur in relation to the aperture. In principle, the instantaneous distance to the aperture can be obtained through stereo vision (if extant). Straightforward geometrical considerations reveal that the width of the aperture can be estimated by combining this distance information with the visual angle subtended by the aperture. In addition, the time to reach the aperture can be estimated from either (a) the rate of change of the distance, or (b) the rate of change of the visual angle, combined with information about the instantaneous distance (from stereo), or (c) the ratio between the visual angle of the aperture and the rate of change of this angle^8^. However, it is believed that most non-predatory birds lack frontal stereo vision, even if there is an overlap between the visual fields of the two eyes^9^,^10^. Therefore, birds such as budgerigars are likely to rely upon cues based on optic flow to extract these variables^2^,^5^. Here we seek to identify the cues that play a critical role in determining whether wing closure is necessary, and in controlling its timing.

A broader question concerns the extent to which birds engage in anticipatory planning of their entire flight, from take off to cruise and finally landing, when they are required to pass through a narrow aperture *en route*. Our reconstruction of the entire 3D trajectories of budgerigars flying through apertures of various widths, and analysis of the height profiles, speed profiles and wing closures reveals a number of unexpected strategies that the birds evidently use to pre-plan their flights—well in advance of encountering the aperture—to ensure a safe transit through it, as well as a smooth subsequent landing.

## Methods

### Ethics Statement

All experiments were carried out in accordance with the Australian Law on the protection and welfare of laboratory animals and the approval of the Animal Experimentation Ethics Committees of the University of Queensland, Brisbane, Australia.

### Subjects

Seven adult male budgerigars (*Melopsittacus undulatus*) aged between three and five years old were used in the experiments. They were purchased from a local pet shop at the age of approximately one month and were housed in an outdoor aviary measuring 4 m in length, 2 m in width and 2.2 m in height. The mesh walls of the aviary provided a natural diurnal light cycle. Perches were placed in areas with regular sunlight as well as in more sheltered areas shielded from inclement weather. The birds were transported to the experiment site at The University of Queensland’s Pinjarra Hills field station about two to three times a week. They were kept in groups of up to four in smaller cages (47 × 34.5 × 82 cm) for a period not exceeding eight hours for each trip. The birds were delivered back to the aviary at the end of each experimental day.

### Apparatus and implementation

The experiments were set up in an indoor tunnel of dimensions 7.28 m (length) ×1.36 m (width) ×2.44 m (height). It comprised white walls, a white ceiling, and a grey floor. The front and back end walls were covered with black cloth to standardise visual cues. A vertically oriented, slit-like aperture was placed in the middle of the tunnel by suspending two panels of cloth from the ceiling all the way to the floor. These panels were decorated with a checkerboard pattern to make them clearly visible.

Eight different aperture widths were tested, varying from 24 to 38 cm in steps of 2 cm. This range of aperture widths bracketed the birds’ wingspans, which varied from 29–33 cm depending upon the individual bird. Accordingly, the relative difference between the aperture width and the birds’ wingspan varied from −5 to +5 cm. Additionally, the birds were also tested in an ‘extreme narrow aperture’ condition with an aperture width of 13 cm (−16 to −20 cm relative to the birds’ wingspan), as well as a control condition where no panels were present. In the results, the aperture widths are represented as relative widths (i.e. as the difference between actual width of the aperture and the wingspan of the bird in question).

### Training

The birds were initially trained to take off from a perch at one end of the tunnel and fly through the aperture to reach the other end of the tunnel for no more than five times before their flights were recorded. The take-off perch was either held by an experimenter or was affixed to the wall. A birdcage, 50 cm high, containing familiar birds was placed on the floor at the other end of the tunnel. This motivated the experimental bird to take off from its perch, fly through the aperture and land on the birdcage.

### Recording

The flights were recorded using a high-speed camera (DRS data & imaging systems, Inc., Oakland, NJ). Camera operation and video acquisition were controlled by special-purpose software MiDAS 2.0 (Xcitex, Inc., Cambridge, MA). A downward-looking camera was mounted from the ceiling, 50 cm in front of the aperture. Flights were recorded at 200 frames per second, which provided adequate temporal resolution for investigation of the wing beat cycle during the flight and the passage through the aperture. For each of the seven birds, ten flights were recorded in each experimental and control condition, summing up to a total of 560 flights.

### Reconstruction of 3D aerial trajectories

The video of each flight was initially processed using a purpose-written Matlab program that computed inter-frame image differences to locate the position of the bird in each frame. From this image sequence, we manually digitised the pixel locations of the two wing tip positions of the bird in those frames where the wings were fully extended (which we denote as W_ex_ frames). These pixel locations in the camera image were projected on to a calibration grid on the floor to obtain the locations of the projected wingtips on the floor. The length of the projected wingspan was then combined with the actual wingspan, measured for each individual bird, to compute the height of the bird above the floor at the moment of wing-extension. We assumed that height would change only marginally between successive wing-extensions. Thus, we used a linear interpolation of height between successive wing extensions, in combination with the X and Y coordinates of the bird’s centroid, as derived from each frame using an automated tracking approach, to determine the exact 3D position of the bird in the tunnel (note that linear interpolation between W_ex_ frames was only applied to the height data; the X and Y co-ordinates of the bird’s centroid were available for every frame). This automated tracking procedure (which is described and evaluated in greater detail in the SI) delivered samples of the 3D position of the bird at the 200 Hz frame rate of the video camera. The precision of 3D localisation was estimated to be 21 mm ×6 mm ×25 mm, where the numbers represent standard deviations of the error along the X, Y and height directions respectively (see SI for details). These errors are only slightly less precise than stereo reconstruction approaches using two cameras, see e.g.^5^. The volume of this error parallelopiped is 0.00000035% of the measurement volume of the video camera (8.96 m^3^). The calibration grid on the floor was used only during the camera calibration, and was not present in the experiments. Twenty-four flights were excluded from the analysis, for one or more of the following reasons: (a) the bird flew extremely high, so that the majority of the flight was outside the field of view of the camera; (b) flapping flight could not be observed in the flight; (c) the bird did not resume flapping flight after passing through the aperture; or (d) there was an error in the tracking process. Hence, we were able to compute the 3D trajectories for 536 of the total of 560 flights.

For each flight, we computed the 3D speed profile (3D distance travelled between successive frames, divided by the inter-frame interval), the forward speed profile (axial distance travelled between successive frames, divided by the inter-frame interval), and the height profile. To facilitate visualisation and interpretation, the profiles were plotted on a distance scale (with a resolution of 0.1 m), by appropriate interpolation of the respective time series.

### Statistical analysis

The mean profiles for the 3D speed, forward speed and height were computed separately for each bird and aperture condition (as well as the control condition) by averaging across ten flights of each bird per condition. The number of statistically independent samples obtained for each experimental condition was therefore equal to seven, the total number of birds. For certain analyses—where applicable, and as indicated—these bird profiles were then averaged to obtain a mean profile for each aperture.

To facilitate comparison across different experimental conditions, the speed and height at which the birds approached the aperture was measured by averaging the flight over an ‘approach’ window that extended from a point 1.2 m ahead of the aperture (at which point the take-off phase was complete, as indicated by the speed and height profiles) to the plane of the aperture. With the resolution of 0.1 m, the total number of sample points within the approach window is 13. For statistical analysis of the flight profiles (speed and height), we used Aligned Rank Transformed ANOVA using a linear mixed effects model (ART^11^). The ART does not require the data to have a normal distribution. Details of the results of these tests, including tests for homogeneity of variance, and the values of the residuals, are given in the SI. For post hoc comparison, we employed least squared means using the Tukey method for multiple comparisons. To compare the heights of flights that did or did not involve wing closure at various positions along the flight trajectories, we used pair-wise one tailed t-tests.

We also analysed separately the flights in which the birds closed their wings prior to traversing the aperture, and flights in which the birds flew through the aperture without closing their wings. For the flights in which wing closure occurred, we measured the time and the distance to the aperture from the point of the last wing extension just before the aperture. These will be referred to as the *times and distances of wing closures*, denoting the *commencement* of the wing closure, which is assumed to occur at the point of the final wing extension prior to entering the aperture. The point of resumption of the wing beat cycle was taken to be the point at which the wings were fully extended for the first time after the passage through the aperture.

In order to determine whether birds used the distance to aperture or the time to aperture as a cue to initiate their wing closure, we compared the coefficients of variation (CV) of the two variables (measured at the point of initiation of the wing closure) using ART (for other details see above). If the CV of the distance is smaller than the CV of the time, it would imply that distance is more likely to be the important cue, because it exhibits a smaller variability; and vice versa. This measure of dispersion has been previously employed to compare the relative importance of various cues that could potentially trigger landing in flies and birds^7^,^12^.

## Results

In an earlier study, we found that budgerigars flap their wings continuously when passing through an aperture that is wider than their wingspan. On the other hand, the birds close their wings when the aperture is narrower than the wingspan^2^. Videos of birds flying in an aperture-free tunnel, and passing through wide and narrow apertures, are given in the SI (SV1–SV3). How do the birds determine whether wing closure is necessary, when do they make this determination, and where or when does the wing closure occur?

### Position and timing of wing closures

**Figure 1:**
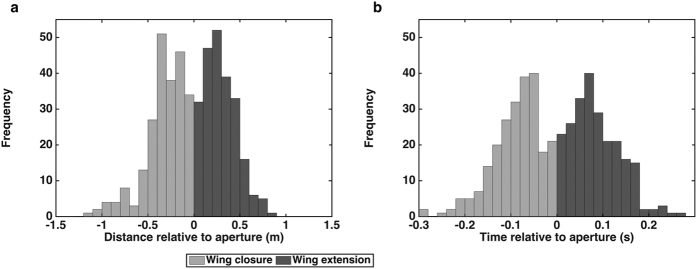
(**a,b)** Histograms of distances (**a**) and times (**b**) of wing closures (light grey), and resumption of flight (dark grey), accumulated over all of the flights that were associated with wing closures.

To investigate these questions, we began by computing histograms of the distances (Fig. 1a) and times (Fig. 1b) of wing closure prior to entering the aperture (light grey bars) and of resumption of the wing beat cycle after passing the aperture (dark bars), for all of the flights in which wing closure was observed. Table 1 displays the mean, SD and CV of the distances and times of the wing closures and the resumption of the flight after passage through the aperture, for various aperture widths. Using ART with the birds as the random factor, and aperture width, wing closure or wing extension, time or distance as the fixed factors, we find that the CV of the wing closure distance (0.516) is similar to that of the wing closure time (0.482) and the two values are not significantly different (ART: F_(1,152)_ = 0.153; P = 0.696). There is no significant difference between the CVs for wing closure and wing extension (ART: F_(1,118)_ = 1.608; P = 0.207). There were indications that there is a significant dependence of the CV of time and distance on the aperture width (ART: F_(6,118)_ = 2.426; P = 0.030), but post hoc analysis revealed that significant differences are few and non-systematic (see SI for details).

Table 1 also shows the corresponding statistics for the distances and times of resumption of the wing beat cycle after the aperture has been traversed. Again, the CV of the distance (0.566) is close to that of the time (0.594), and the two values are not significantly different (see above). We may therefore conclude that the trigger that causes wing closure prior to passage through the aperture is equally likely to be the distance to the aperture, or the time to the aperture. Similarly, the trigger that causes the resumption of flight after passage through the aperture is equally likely to be the distance travelled beyond the aperture, or the time elapsed after passing through the aperture. We shall discuss the implications of these findings later below.

### General properties of all flights

**Figure 2:**
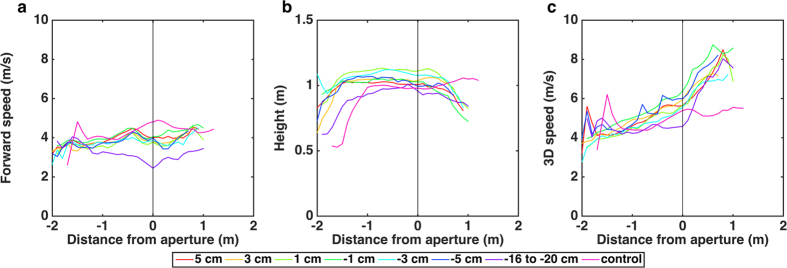
(**a–c)** Profiles of forward speed (**a**), height (**b**) and 3D speed (**c**) for each aperture width and for the control condition, averaged over all flights and birds. Distances are shown relative to the position of the aperture (0 m).

In addition, we examined a number of other characteristics of the birds’ flights by calculating average profiles of the forward flight speed, 3D speed and height. The results are shown in Fig. 2 for each aperture width, as well as for the control condition. Distances are shown relative to the position of the aperture (0 m). Visual inspection reveals that the flight profiles are more or less similar for all apertures, except for the narrowest aperture (−16 to −20 cm), and the control condition.

For all flights and all apertures (excluding the extreme narrow aperture and the no aperture flights), after completion of the take-off phase, the birds flew at a more-or-less constant average forward speed of 3.92 ± 0.20 m/s (as measured over the approach window, see ‘Methods’). The approach speeds for various aperture widths were very similar. ART tests were conducted for all apertures (including the extreme narrow aperture) to examine the effects of aperture width on forward speed, 3D speed and height over the approach window. For this analysis we treated the individual birds (7 total) as the random factor, and the aperture width (8, including the no aperture control condition) and the 13 positions within the approach window as the fixed factors. The analysis revealed a significant effect of aperture width on forward speed (F_(7,615)_ = 48.732; P < 0.001), 3D speed (F_(7,615)_ = 19.203; P < 0.001) and height (F_(7,615)_ = 9.750; P < 0.001). Post hoc comparison revealed that the birds displayed no significant differences in speed across the −5 to 5 cm aperture range, however, there was a significantly higher forward speed in the control condition (4.24 ± 0.31 m/s) compared to all conditions except the −1 cm condition (details in SI). The birds’ speed in the extremely narrow aperture was significantly lower (3.04 ± 0.25 m/s) compared to all other aperture conditions. The birds tend to fly faster in the control condition while they tend to fly slower in the narrowest aperture. The height profiles (Fig. 2) show the expected increase in height during the take-off phase, followed by a period where birds maintain a more-or-less constant cruising height until they reach the aperture. Beyond this point the height begins to decrease, partly because the birds close their wings prior to entering the aperture in some of the flights, and partly because of the initiation of the landing process.

### Properties of flights associated with wing closure

**Figure 3:**
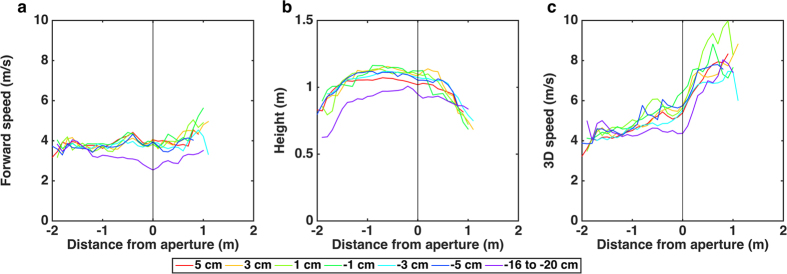
(**a–c)** Profiles of forward speed (**a**), height (**b**) and 3D speed (**c**) for the flights that were associated with wing closure. The profiles are shown for each aperture width, and are averaged over all wing-closure flights and birds. Distances are shown relative to the position of the aperture (0 m).

Analysis of the profiles of the wing closure flights within the approach window reveals no systematic significant dependence on aperture width for height, forward speed or 3D speed for the +5 cm to −5 cm aperture widths (see above, details in SI). However, the narrowest gap is distinctly different from the rest of the apertures – the forward speed (F_(6,462)_ = 33.579; P < 0.001), 3D speed (F_(6,462)_ = 12.119; P < 0.001) and height (F_(6,462)_ = 10.049; P < 0.001) associated with the narrowest gap are significantly lower than for the flights through the other apertures. Excluding the data for narrowest gap, the forward approach speed (3.88 ± 0.21 m/s, Fig. 3a), the 3D approach speed (5.12 ± 0.44 m/s, Fig. 3b) and the height (1.1 ± 0.02 m, Fig. 3c) for all of the other apertures are more or less constant.

The average profiles for all of the flights in which wing closure occurred prior to passage through the aperture are shown in Fig. 3. Comparison of Fig. 3 with Fig. 2 reveals some distinctive characteristics of the flights that involve wing closure. When the birds come close to the aperture, the forward speed begins to drop slightly. One reason for this could be the closure of the wings, which would cause the thrust to drop to zero. Active braking by the wings is unlikely, as there is no direct evidence for this in the videos (see SI: SV1–SV4). The small reduction in forward speed continues until a point just beyond the aperture, after which it increases steadily. This increase in forward speed is most likely triggered by the resumption of flapping flight. Beyond the aperture the height begins to drop rapidly, reflecting the onset of the landing manoeuvre. This causes the 3D speed to increase rapidly, prior to touchdown. The interpretation of the behaviour displayed in the data of Fig. 3 can be confirmed and visualised more directly by replotting the profiles in relation to the point of wing closure, rather than the position of the aperture. This is illustrated in the SI.

**Figure 4:**
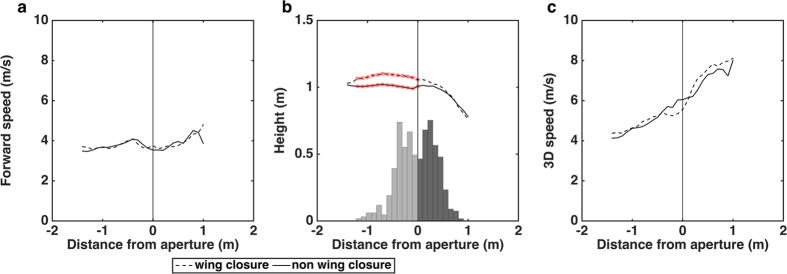
(**a–c)** Profiles of average forward speed (**a**), average height (**b**) and average 3D speed (**c**) for all wing closure flights (dashed line) and all flights that were not associated with wing closures (solid line). Points of significant difference are marked by red symbols in (**b**). Panel **b** includes the histograms of the distributions of the positions of wing closure (light grey bars) and of resumption of flight (dark grey bars).

Figure 4 shows a comparison of the profiles of forward speed, height and 3D speed for the wing closure flights, with the corresponding profiles of the flights that were not associated with a wing closure, averaged over all birds and aperture widths (including the narrowest aperture). Also shown are the histograms of the distributions of the positions of wing closure (light grey bars) and of resumption of the flight (dark grey bars). Judging from the graph profiles, there is no substantial difference between the two categories of flights in the profiles for forward speed (Fig. 4a) and 3D speed (Fig. 4c) at any position along the flight. This means that, even when the wings are temporarily closed, the birds ‘projectile’ through the aperture at about the same speed as when they pass through without interrupting their wing beat cycle. Both groups of flights also display a consistent increase in the 3D speed after passage through the aperture (Fig. 4c), reflecting the increase in the descent speed in preparation for landing. General analysis using ART, with the birds as the random factor and aperture width and wing closure or non-wing closure as the fixed factors. The birds fly significantly higher in flights in which they close their wings (ART: F_(1,44)_ = 7.944; P = 0.007). Detailed analysis of the trajectories reveals that in the range of −1.2 m to 0 m, the birds fly at a significantly greater height in the flights in which they close their wings, as compared to flights in which they do not interrupt their wing beat cycle (one tailed T-test, p < 0.05; see red highlights, Fig. 4b). The height profiles of the two groups of flights depart significantly from each other at a distance of 1.2 m from the aperture. This point is well ahead of the mean point of wing closure (which is at 0.37 ± 0.18 m, Table 1). The height profiles reunite slightly beyond the point of resumption of the flight (at 0.28 ± 0.16 m).

## Discussion

Our findings reveal, for the first time, that birds display sophisticated planning in preparation for flight through narrow passages: many properties of the approach trajectory depend not only upon the presence of an oncoming aperture, but also upon whether or not they will close their wings when they reach it. In the control condition (in which there was no aperture) the birds flew at a relatively low height and a significantly higher speed, compared to all of the conditions in which an aperture was present. Presumably, flying slower when negotiating an aperture ensures a safer passage. In an earlier study, we showed that budgerigars are very precise at estimating aperture width from a distance^2^: wing closure occurs only when the aperture is narrower than the wingspan by at least 1 cm^2^. Subsequent investigations^3^,^4^ have also reported anticipatory behaviour in pigeons, which lower their flight speed at a comparable distance of about 1.5 m prior to entering a densely cluttered environment.

What are the visual cues that the birds could use to decide, from a distance, whether an aperture is narrow enough to require closure of the wings ? One potential cue would be stereo vision. Binocular overlap of the visual fields is present in a range of predatory birds such as owls and hawks^9^,^10^, as well as some non-predatory birds such as crows^13^, certain forest passerines^14^ and pigeons^15^. However, the presence of binocular overlap does not necessarily imply the existence of stereo vision. Stereo capability has been demonstrated rigorously in the falcon^16^, the owl^17^ and the pigeon^15^. Some authors have suggested that non-predatory birds that possess stereo vision use this capacity only for close-range tasks such as pecking for food^14^ or tool manipulation^13^, and not for long- range distance perception^9^,^10^. Putting this caveat aside, and turning to our experiments, if a bird were to possess long range stereo vision, it could register the distance to the aperture at any point along its approach. This distance information could then be combined with information on the instantaneous visual angle subtended by the aperture to determine the aperture width, and thus decide whether wing closure would be necessary.

Another means of estimating aperture width would be through information derived from optic flow. The data of Figs 2 and 3 reveal that, for all apertures but the narrowest one, the birds approach the aperture at a more or less constant speed. During the approach the image of each edge of the aperture would move at a progressively greater rate in the retina, and this rate would depend upon aperture width. Consequently, the width of the aperture could be calibrated directly in terms of the time course of the optic flow that is generated by the edges of the aperture, as described in detail in the SI.

Let us now turn to a second question: If the aperture is narrower than the wingspan, and wing closure is deemed necessary, where (or when) does the wing closure occur, and what is the cue that initiates this closure ? Our results indicate that the wing closure, if it occurs, is initiated at a mean distance of 37 cm from the aperture, and at a mean time of 100 ms before entering it, irrespective of aperture width (Table 1). Is the initiation of wing closure triggered at a critical distance, or a critical time? Our analysis reveals that the coefficients of variation (CV) of the distances and times are very similar (Table 1), and not statistically different. This indicates that the triggering of the wing closure (and re-opening) can depend equally legitimately on distance or time. This makes sense, because our observation of a constant approach speed of ~3.92 m/s for all of the apertures (except for the narrowest one) implies that initiating wing closure at a mean distance of 37 cm to the aperture (Table 1) is exactly equivalent to initiating it at a mean time to the aperture of (0.37/3.92) = 0.094 s. This figure is very close to the observed mean time of 0.099 s (Table 1). The distances and times of wing re-opening also show similar coefficients of variation, again supporting the notion that, if the approach speed is constant, the computation of time and distance are equivalent. In the SI we demonstrate mathematically that, if the approach speed is constant, the CV of distance should be equal to the CV of time. It is likely, however, that the re-opening of the wings is orchestrated to occur at a specific *time* past the transit, rather than a specific *distance*. This is because, after transit, the aperture will probably be occluded by the body and the wings, making the participation of visual cues unlikely.

What are the sensory cues that could initiate the closure of the wings? Again, stereo vision is one potential candidate. Given that the wing closure occurs at an approximately constant distance of 37 cm to the aperture, it is only necessary to continuously monitor the distance to the aperture and initiate wing closure when this distance drops below 37 cm. Optic flow cues can also provide the requisite information, and they can be used in two different ways to solve this particular task. One method would be to take advantage of the fact that the approach speed is constant over time, independent of aperture width, and has a ‘known’ value. Given this, it is only necessary to track the angular velocity of the image of one edge of the aperture during the approach, and to initiate the wing closure when the angular velocity of the edge has exceeded a prescribed threshold value for the direction in which that edge is currently viewed. These prescribed values are determined from the ‘known’ constant approach speed, and can be incorporated in the nervous system as pre-set thresholds. Details are given in the SI. Another method would be to exploit the fact that computing the distance to the aperture is equivalent to computing the time to reach the aperture, because of the constancy of the approach speed. The work of Lee and colleagues^6^ has shown that birds are capable of computing the time to contact a surface that is being approached, without knowledge of the speed of approach or the distance to the surface. In our context, the time to reach the aperture (TRA) would be given by the ratio of the visual angle subtended by the aperture, to the rate of its expansion (see SI for details). TRA can be computed continuously, to initiate wing closure when its value drops below 100 ms.

The constancy of approach speed that is displayed by the budgerigars in our experiments, as well as in other studies (e.g. Schiffner and Srinivasan^18^ and unpublished data) allows optic flow cues to be used in a simpler and more direct fashion, compared to situations when the speed of flight is unknown and variable. Once the flight speed is fixed and ‘known’, optic flow information can be used in a very direct and straightforward way to calibrate the distances to objects, as well as their sizes.

In contrast to budgerigars, insects fly at a wide range of different speeds, thus making the use of optic flow cues more complex and challenging. This is because the absolute distance or size of an object in the environment cannot be inferred without knowledge of the speed of flight. Whether insects resolve this problem, and if so, how they do it, continues to be an enigma, at least in some contexts (e.g. Srinivasan^19^). Our data do not allow us to decide definitively whether budgerigars use cues based on stereo, or optic flow, or a combination of both kinds of cues, to determine whether and when to close their wings. However, the considerations presented here and in the SI indicate that optic-flow cues can, at least in principle, provide all of the information that is needed to determine (a) whether wing closure is necessary, and (b) if so, when (or where) this closure should be initiated. This is possible because the birds approach the apertures at a constant speed, irrespective of the aperture width.

Our study also reveals that, among all of the conditions in which an aperture was present, the birds flew significantly higher in the flights in which they closed their wings. This appears to be a pre-compensation for the loss of height that results from wing closure, which temporarily eliminates lift force. The height trajectories for the wing closure and non-closure flights are virtually identical up to a point 1.2 m ahead of the aperture. The trajectories depart from each other beyond this point, indicating that the decision to close the wings occurs here, if not earlier in the flight. This location is well ahead of the actual point of initiation of wing closure, which occurs at a mean distance of 0.37 m from the aperture. Thus, the birds ascertain the necessity for wing closure at a considerable distance from the aperture - well ahead of the point at which they initiate the wing closure.

For the wing closure flights, the height trajectories reunite after the point of resumption of the wing beat cycle for the wing closure flights (Fig. 4b). Beyond the point of flight resumption, the two trajectories are again identical. This implies that the wing closure flights are pre-planned carefully and precisely to ensure that the birds gain height well before commencing the wing closure, and also to ensure that they subsequently follow the same landing trajectories as the non-closure flights. The question of whether this planning is conscious or pre-programmed is, of course, an interesting and unresolved issue that merits further investigation.

In conclusion, our findings reveal that budgerigars plan their flights through narrow apertures in at least two ways. First, the birds are able to assess the width of the aperture in relation to their wingspan well ahead of their passage through the aperture—at a distance of at least 1.2 m away—and they close their wings, if necessary, at a more-or-less constant distance of 37 cm from the aperture, regardless of aperture width. Second, the flights that are associated with wing closure reveal the existence of a rather elaborate and sophisticated pre-planning of the entire flight trajectory. When a bird plans a wing closure it flies to a greater height, well in advance of the aperture, in order to compensate for the loss of height that is caused by the wing closure, and also to ensure that the final phase of the flight—the landing—traces the same trajectory as in the flights in which it does not close its wings. Evidently, the birds strive to ensure that the landings always follow a constant, safe trajectory, irrespective of the manoeuvres that are performed mid-flight.

## Additional Information

**How to cite this article**: Vo, H. D. *et al.* Anticipatory Manoeuvres in Bird Flight. *Sci. Rep.* **6**, 27591; doi: 10.1038/srep27591 (2016).

## Supplementary Material

Supplementary Information

Supplementary Video S1

Supplementary Video S2

Supplementary Video S3

Supplementary Video S4

## Figures and Tables

**Table 1:** Analysis of the point of wing closure before the aperture and the point of flight resumption after the aperture, in distance (m) and time (s).

Aperture Width	*Time (s)*		*Distance (m)*	
	Mean +/−SD	CV	Mean +/−SD	CV
*WING CLOSURE*				
**5**	−0.101 +/−0.044	0.501	−0.385 +/−0.187	0.534
**3**	−0.089 +/−0.038	0.437	−0.335 +/−0.141	0.412
**1**	−0.151 +/−0.068	0.436	−0.562 +/−0.256	0.406
**−1**	−0.114 +/−0.043	0.534	−0.446 +/−0.231	0.703
**−3**	−0.091 +/−0.036	0.401	−0.338 +/−0.131	0.403
**−5**	−0.085 +/−0.042	0.520	−0.336 +/−0.186	0.561
**−16 to −20**	−0.058 +/−0.031	0.547	−0.154 +/−0.092	0.593
**Average**	**−0.099 +/−0.043**	**0.482**	**−0.365 +/−0.175**	**0.516**
*WING EXTENSION*				
**5**	0.083 +/−0.031	0.419	0.347 +/−0.132	0.408
**3**	0.058 +/−0.034	0.610	0.238 +/−0.14	0.576
**1**	0.073 +/−0.05	0.732	0.285 +/−0.198	0.665
**−1**	0.043 + /−0.048	0.948	0.159 +/−0.169	0.901
**−3**	0.066 + /−0.038	0.581	0.256 +/−0.14	0.562
**−5**	0.084 +/−0.041	0.441	0.329 +/−0.165	0.449
**−16 to −20**	0.118 +/−0.05	0.427	0.338 +/−0.138	0.401
**Average**	**0.075 +/−0.042**	**0.594**	**0.279 +/−0.155**	**0.566**

The Table shows the means and standard deviations of these measurements, and their coefficients of variation (CV), for various aperture widths.
