# Supplementary material for: Anticipatory Manoeuvres in Bird Flight
Source: Sci Rep. 2016 Jun 8;6:27591. doi: 10.1038/srep27591 (PMC4897778; doi:10.1038/srep27591)
Supplement: Supplementary Information [file srep27591-s1.pdf]

ANTICIPATORY MANOEUVRES IN BIRD FLIGHT

Hong D. Vo; Ingo Schiffner; Mandyam V. Srinivasan

SUPPLEMENTARY INFORMATION

RECONSTRUCTION OF 3D TRAJECTORIES

Our method uses footage from a single, downward facing video camera to reconstruct the flight trajectories in 3D. The wingspan W of the bird is assumed to be known, as is the height H of the camera above the floor.

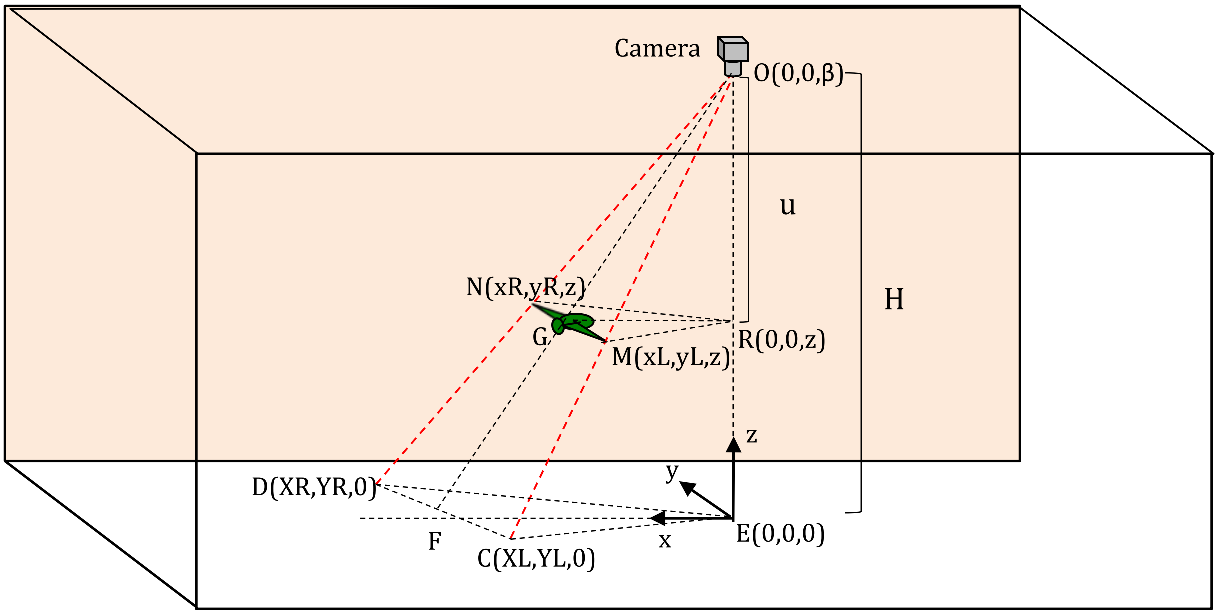

Supplementary Figure S1: Illustration of method for 3D reconstruction of flight trajectories

The procedure for reconstructing a bird's 3D trajectory in the tunnel can be summarised as follows, with reference to Fig. S1:

a) A calibration grid is laid out on the floor (grey lines, Fig. S1), in which the co-ordinate position (X,Y,Z) of each of the grid corners is known with respect to an arbitrarily chosen origin on the floor which can be, for example, the point directly beneath the camera. (Since the grid is on the floor, Z=0 for all of the grid points).

b) The pixel positions of the grid locations are then determined in the camera image, to obtain a one-to-one mapping between the real co-ordinates of the grid locations on the floor and their corresponding pixel coordinates in the image.
c) Video footage of a bird flying through the tunnel, as captured by the overhead camera, is analysed using custom-written software, to track the pixel position of the centroid of the image of the bird in each video frame. (Details of this procedure are described below).
d) The frames of video sequence in which the wings are fully extended (Wex) are determined, and the wingspan is measured in each of these frames as the pixel distance between the wingtips. (Frame-by-frame inspection of videos of several flights shows no evidence of significant roll in the bird's body, so that the distance between the wingtips in the Wex frames is indicative of the true wingspan of the bird)
e) The positions of the images of the wingtips are referenced to the image of the calibration grid, and these positions are projected to the floor grid (as points C and D in Fig S1) by using a process of 2D interpolation implemented by the Matlab function *TriScatteredInterp*. The wingspan Wf of the projected image on the floor is then computed as the physical distance between the projected positions of the wingtips on the floor.
f) The ratio of the bird's known wingspan, W, to the wingspan Wf of the projected image on the floor (W/Wf), is then equal to the ratio of the vertical distance of the bird from the camera (U) to the height H of the camera above the floor (U/H), by similar triangles. This enables the calculation of U, and thus the height of the bird above the floor, at each of the Wex frames.
g) The height of the bird in all of the other frames is estimated by linear interpolation between the heights computed at the Wex frames. At the end of this step we have an estimate of the height of the bird (Z) for each frame of the video sequence.
h) The position of the centroid of the image of the bird in each video frame [as computed in (c)] is projected on to the floor by using a process of 2D interpolation, as described in (e). The (X,Y) position of the bird in the tunnel at each frame instant is then computed by combining the projected floor position of

the image centroid at with the corresponding height and using the geometry of similar triangles, in a process analogous to that described in (f).

i) The final result is an estimate of the (X,Y,Z) co-ordinates of the bird for each frame of the video sequence.

The calibration grid on the floor was used only during the camera calibration, and was not present in the experiments. The precision of the 3D trajectory reconstruction procedure was evaluated by placing a small test target - a model bird with with a calibrated wingspan of 30 cm - at 44 different, known 3D locations within the tunnel, of which 39 were within the boundary of the grid. The results are shown in Table S1, below. The standard deviations of the errors along the x, y and H directions were 21 mm (x), 6.1 mm (y) and 25.4 mm (H).

Supplementary Table S1: Test of accuracy of 3D position measurements. X, Y and H represent (in cm) the true co-ordinates of a test target along the axial (length), width and height of the tunnel, respectively. X calc, Y calc and H calc are the calculated values of these co-ordinates, and X error, Y error and H error represent the respective errors. The standard deviation of the errors (SD) are given at the end of the table. The 5 missing measurements pertain to target positions whose floor projections fell outside the grid.

Points	X (cm)	X calc	X error	Y (cm)	Y calc	Y error	H (cm)	H calc	H error
1	36.75	-	-	115.5	-	-	91	-	-
2	36.75	-	-	75.5	-	-	91	-	-
3	36.75	-	-	35.5	-	-	91	-	-
4	116.75	114.59	-2.16	115.5	115.12	-0.38	91	89.24	-1.76
5	116.75	116.25	-0.50	75.5	75.25	-0.25	91	92.21	1.21
6	116.75	112.91	-3.84	35.5	35.43	-0.07	91	87.53	-3.47
7	216.75	216.47	-0.28	115.5	115.46	-0.04	91	87.86	-3.14
8	216.75	216.61	-0.14	75.5	75.65	0.15	91	95.20	4.20
9	216.75	216.49	-0.26	35.5	35.94	0.44	91	89.37	-1.63
10	316.75	316.37	-0.38	115.5	115.33	-0.17	91	90.34	-0.66
11	316.75	316.76	0.01	75.5	75.45	-0.05	91	91.88	0.88
12	316.75	318.51	1.76	35.5	34.72	-0.78	91	87.28	-3.72
13	36.75	-	-	115.5	-	-	79	-	-
14	36.75	40.32	3.57	75.5	75.69	0.19	79	80.02	1.02

15	36.75	-	-	35.5	-	-	79	-	-
16	136.75	136.58	-0.17	115.5	115.62	0.12	79	79.13	0.13
17	136.75	138.51	1.76	75.5	75.51	0.01	79	83.09	4.09
18	136.75	136.89	0.14	35.5	36.15	0.65	79	80.10	1.10
19	236.75	234.68	-2.07	115.5	116.37	0.87	79	74.71	-4.30
20	236.75	233.38	-3.37	75.5	76.13	0.63	79	81.48	2.48
21	236.75	235.00	-1.75	35.5	36.42	0.92	79	78.82	-0.18
22	336.75	336.80	0.05	115.5	114.59	-0.91	79	79.99	0.99
23	336.75	333.92	-2.83	75.5	75.25	-0.25	79	82.73	3.73
24	336.75	336.71	-0.04	35.5	35.94	0.44	79	79.15	0.15
25	36.75	40.70	3.95	115.5	114.27	-1.23	65.5	68.99	3.49
26	36.75	39.67	2.92	75.5	75.91	0.41	65.5	68.13	2.63
27	36.75	38.57	1.82	35.5	35.85	0.35	65.5	67.39	1.89
28	136.75	33.08	-3.67	115.5	114.09	-1.41	65.5	61.67	-3.83
29	136.75	138.08	1.33	75.5	74.88	-0.62	65.5	69.27	3.77
30	136.75	138.12	1.37	35.5	35.90	0.40	65.5	67.35	1.85
31	236.75	138.34	1.59	115.5	115.72	0.22	65.5	66.66	1.16
32	236.75	136.17	-0.58	75.5	75.56	0.06	65.5	63.34	-2.16
33	236.75	235.53	-1.22	35.5	34.93	-0.57	65.5	64.44	-1.06
34	336.75	234.92	-1.83	115.5	115.93	0.43	65.5	67.65	2.15
35	336.75	235.61	-1.14	75.5	76.21	0.71	65.5	66.36	0.86
36	336.75	236.73	-0.02	35.5	36.80	1.30	65.5	61.50	-4.00
37	396.75	338.17	1.42	115.5	115.09	-0.41	65.5	64.72	-0.78
38	396.75	336.05	-0.70	75.5	75.66	0.16	65.5	67.88	2.38
39	396.75	338.22	1.47	35.5	36.40	0.90	65.5	65.55	0.05
40	36.75	340.04	3.29	19.5	18.79	-0.71	65.5	61.62	-3.88
41	136.75	392.44	-4.31	19.5	18.66	-0.84	65.5	70.01	4.51
42	236.75	400.11	3.36	19.5	19.27	-0.23	65.5	62.98	-2.52
43	336.75	396.52	-0.23	23.5	23.48	-0.02	65.5	65.51	0.01
44	396.75	395.72	-1.03	23.5	23.11	-0.39	65.5	66.04	0.54
**Averaged Error (cm)**			**-0.07**			**0.00**			**0.21**
**SD Error (cm)**			**2.09**			**0.61**			**2.57**

FLIGHT TRACKING PERFORMANCE: COMPARISON OF AUTOMATIC AND MANUAL TRACKING METHODS

To reduce the intensive labour of manual tracking, we created a purposed-written Matlab program to automate the tracking of the centroid of the birds' image. This was accomplished by generating pixel-wise absolute difference images between adjacent

frames, and computing the position of the centroid of this image to track the movement of the bird's centroid. To evaluate the accuracy and robustness of this automated tracking procedure, the resulting trajectories were compared with those obtained from a few manually tracked flights. An example of one flight is illustrated in Figs. S2-S4. We manually digitised the position of the head, because it is a highly visible part of the bird and displays a stable, horizontal orientation that is largely independent of body roll. The trajectory of the same flight was then constructed by using the pixel position of the head, rather than the bird's centroid, to define the position of the bird.

Figure S2 shows a plan view of a comparison between the automatically tracked trajectory of the birds' centroid (green) and the trajectory obtained by manually tracking the bird's head (blue). The two trajectories are in good agreement. Figure S3 shows a comparison of the height profiles and Fig. S4 shows axial (forward) speed profiles for the same flight as in Fig. S2, using automated centroid tracking (green), and manual tracking of the head (blue). The auto-tracked axial speed trajectory displays an oscillation because it calculates the centroid of the bird in each frame, which is influenced by the front-back motion of the wings. This causes apparent oscillations in the position as well as the speed of the bird along the flight direction (x). We used a 9-point average filter to eliminate these spurious speed oscillations. The result of this filtering, shown by the red curve, corresponds closely to the profile obtained using manual tracking of the head (blue).

Overall, it is clear from Figs. S2-S4 that the results obtained from the automated tracking of the centroid are in close agreement with those obtained by manual tracking of the head (which should yield more accurate results), when the spurious, wing-induced speed oscillations generated by the wing motions are filtered out wherever necessary. It is evident that this filtering is required only when analysing the speed profile.

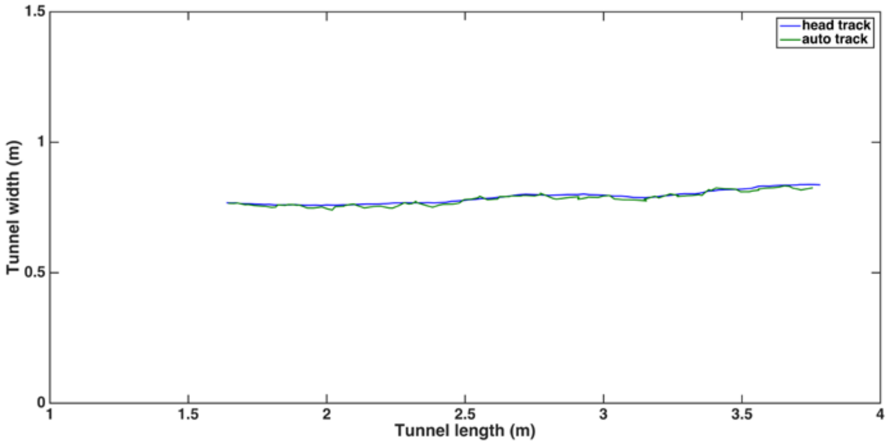

Supplementary Figure S2: Comparison of plan views of a flight trajectory tracked using the automated centroid tracking (green) and manual tracking of the head (blue).

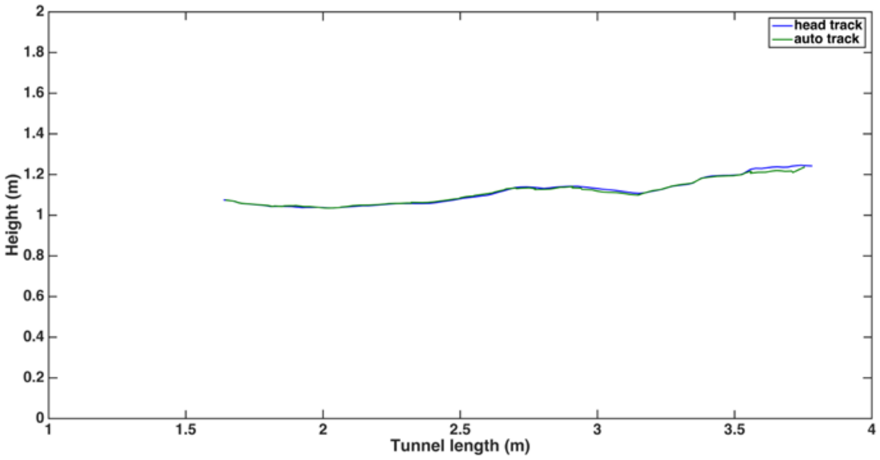

Supplementary Figure S3: Comparison of height profiles for the same flight as in Fig. S2, using automated centroid tracking (green) and manual tracking of the head (blue).

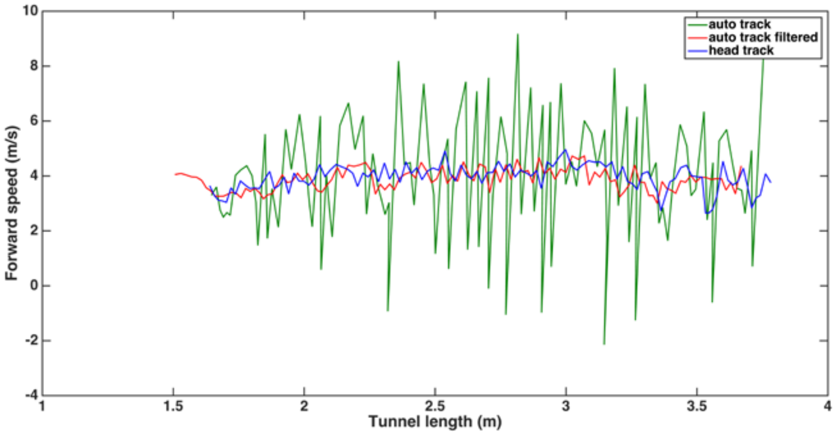

Supplementary Figure S4: Comparison of axial (forward) speed profiles for the same flight as in Fig. S2, using automated centroid tracking (unfiltered: green; filtered: red) and manual tracking of the head (blue). Application of the filter suppresses the spurious wing-induced oscillations of speed that are generated by the automated tracking.

FLIGHT PROFILES REPLOTTED IN RELATION TO DISTANCE FROM APERTURE

The interpretation of the behaviour displayed in the data of Fig. 3 can be confirmed and visualised more directly by replotting the profiles in relation to the point of wing closure, rather than the position of the aperture. The replotted profiles are shown in Fig. S5, where 0 m represents the point of wing closure. In Fig. S5a, we observe that the forward speed begins to decrease at a slow rate, and by a small amount immediately after wing closure. The height (Fig. S5b) begins to drop immediately after the point of wing closure, as surmised for the data in Fig. 3b. The 3D speed (Fig. S5c) starts to increase immediately after the point of wing closure, as surmised for the data in Fig. 3c because the reduction in height implies an increase in the vertical speed.

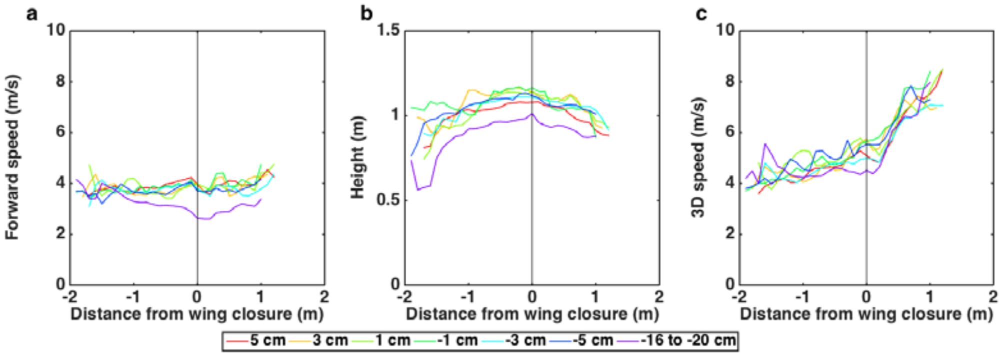

Supplementary Figure S5: Average forward speed (a), height (b), and 3D speed (c) for each experimental condition of trials during which birds closed their wings, shown as relative distance to the point of wing closure (0 m).

SUPPLEMENTARY VIDEOS

Four examples of the flights of one bird (bird *Four*) are shown in videos SV1, SV2, SV3 and SV4. Clip SV1 depicts the flight of the bird in the tunnel when there is no intervening aperture. In SV2, the bird passes through an aperture that is 5 cm wider than its wingspan (+5 cm) without closing its wings. In SV3, the bird passes through an aperture

that is 5cm narrower than its wingspan (-5 cm), and closes its wings before entering the aperture. Clip SV4 shows a side view of the same bird while landing on a perch, illustrating its behaviour during active braking.

A reliable and easily detected indication of active braking before entering the aperture is an increase in the pitch of the body (a lowering of the abdomen and the tail). This is clearly evident in the landing video SV4. In downward-looking views of bird flight, the increased pitch would up as a shortening of the projected body length. By this criterion, there is no evidence of active braking in any of the videos SV1-SV3.

Video SV1: Video of a bird (*Four*) flying in an aperture-free tunnel
Video SV2: Video of *Four* passing through an aperture that is 5 cm wider than his wingspan.
Video SV3: Video of *Four* passing through an aperture that is 5 cm narrower than his wingspan.
Video SV4: Video (side view) of *Four* while landing on a perch.

SUPPLEMENTARY DISCUSSION

Relationship between the coefficients of variation of distance and time to initiation of wing closure

If the birds approach the aperture at a constant speed V, then wing closure will be initiated at a distance D that is inversely proportional to the time T to the aperture. That is,

$$D = V/T, \qquad (1)$$

Which can be rearranged to read

$$DT = V \qquad (2)$$

where V is a constant. We use perturbation analysis to determine the relationship between small variations in D and T. These variations reflect noise in the behavioural response, which can arise from noise at several levels of the sensorimotor pathway.
Perturbing equn (2), we obtain

$$\Delta D.T + \Delta T.D = 0 \qquad (3)$$

Dividing both sides of (3) by D.T and rearranging, we obtain

$$\frac{\Delta D}{D} = -\frac{\Delta T}{T} \qquad (4)$$

If we consider only the magnitudes of the perturbations, we can drop the (-) sign and write

$$\frac{\Delta D}{D} = \frac{\Delta T}{T} \qquad (5)$$

We observe that the left hand side of (5) is a measure of the coefficient of variation of D, while the right hand side is a measure of the coefficient of variation of T. Thus, when the approach speed is constant, we would expect the CV of distance to be equal to the CV of time. This is exactly what we find in our experiments, which reinforces our observation that the approach speed is constant.

Determining whether to close the wings, and if so where (or when)

Consider a bird, flying at a speed V, approaching an aperture of width W (Inset, Fig. S3). In this analysis we assume that the flight speed is constant over aperture distances that ranges from 2 m (well ahead of the point of judgement of aperture width, estimated to be at 1.2 m) to 0 m (beyond the point of initiation of wing closure), This is a reasonable approximation (see Figs. 3a, 4a). For a constant flight velocity, it can be shown that the angular velocity ω of the image of an edge of the aperture in the eye will be:

$$\omega = \frac{2V}{W}\sin^2\theta \qquad (1)$$

where θ is the viewing direction of the edge. It can also be shown that, when the bird is at a distance D from the aperture, the angular velocity of the image of edge of the aperture in the eye will be:

$$\omega = \frac{V}{2D}\sin 2\theta \qquad (2)$$

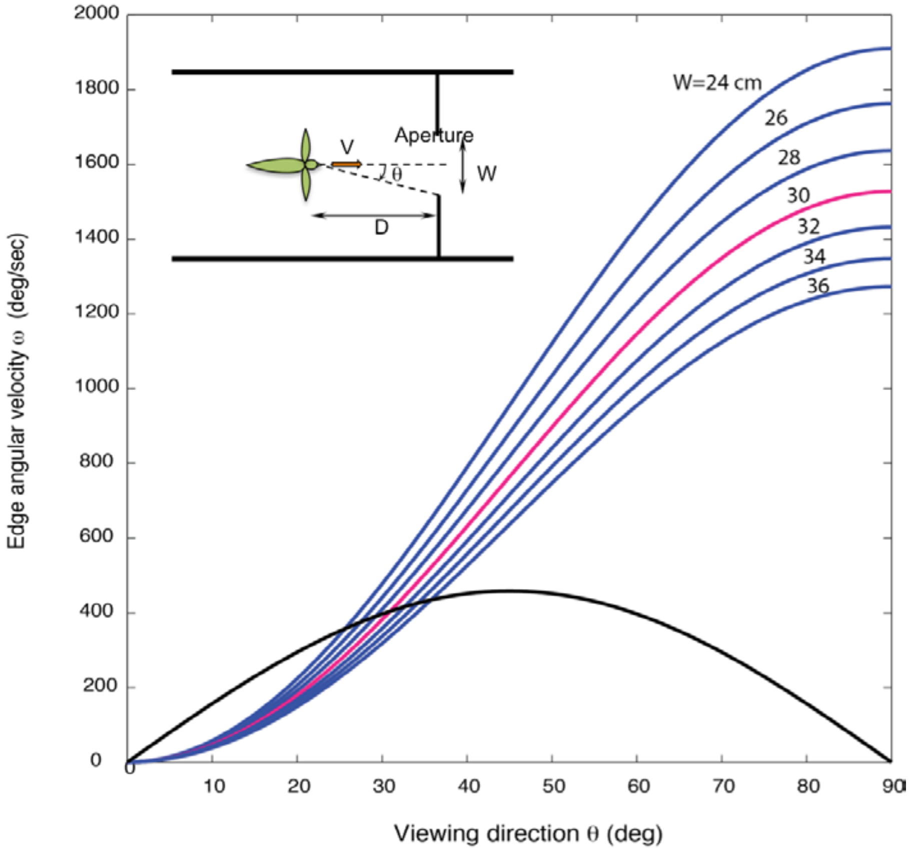

Supplementary Figure S3. Inset: Overhead view of a bird approaching an aperture of width W at a speed V. D is the instantaneous distance to the aperture. The curves show the expected variation of the angular velocity ω of an edge with the viewing direction θ during approaches at a speed of 4.0 m/s toward gaps of various widths W ranging from 24 cm to 36 cm (blue), with the 30 cm width highlighted in red. The back curve shows the expected variation of edge angular velocity as a function of viewing angle when the bird is 25 cm from the gap.

The plots in Fig. S3, based on equation (1), show how ω can be expected to vary with the viewing direction θ of the edge as the aperture is approached at a speed V= 4.0 m/s for various aperture widths W varying from 24 cm to 36 cm. If the approach speed is constant and independent of aperture width, this relationship provides a simple means by which the width of the aperture can be estimated by the approaching bird. For example, consider a bird with a wingspan of 30 cm. For an aperture of width 30 cm, as highlighted by the red curve in Fig. S3, the angular velocity ω of the image of the edge

would be 300 °/s when the edge is viewed at 20°, 750 °/s when the edge is viewed at 45°, and so on. If the values of ω are greater than the prescribed values in the corresponding viewing directions, this would imply that the aperture is narrower than the bird's wingspan, and that the bird should close its wings. Thus, if a bird with a wingspan of 30 cm carries a representation of the red curve in its visual system in the form of a set of expected angular velocities in various viewing directions, then the visual system would signal that the aperture is narrower than the wingspan if the observed ω generated by the edge when it is in any viewing direction θ exceeds the expected value for that direction.

The accuracy of the decision to close the wings can be enhanced by monitoring ω not just in a single viewing direction but over a series of progressively increasing viewing angles, and comparing the measured ω values with the corresponding expected ω values. Another way of improving the reliability of the decision would be to integrate, over time, the measured values of ω over a prescribed range of viewing directions, and compare this result with the expected value of this integral for a 30 cm aperture. If the measured value of the integral is greater than the expected value, the aperture is narrower than 30 cm, implying that wing closure is necessary; if it is lower than the expected value, the aperture is wider than 30 cm and does not require closure of the wings. We note that these computations would have to be performed ahead of the point where the necessity for wing closure is determined, i.e. at distances greater than 1.2 m from the aperture.

If wing closure is required, the relationship in equation (2) can also be used to ensure that this occurs at a prescribed distance D_0 from the aperture. When the bird is at a distance D_0 from the aperture, the expected angular velocity ω of the image of the edge (as a function of the direction in which the edge is viewed) would be given by

$$\omega = \frac{V}{2D_0}\sin 2\theta \qquad (3)$$

The black curve in Fig. S3 shows the profile of the expected value of ω for various viewing directions θ when the bird is at a distance D_0 = 0.37 m from the aperture. When

the measured value of ω exceeds the expected value for the current direction θ of the edge, the distance to the aperture has dropped below 0.37 m.

An alternative strategy would be to initiate wing closure at a specific time T_0 before reaching the aperture. For an approach velocity V, the time T to reach the aperture would be D/V when the bird is at a distance D from it. Setting $T=D/V$, we may rewrite (2) as

$$T = \frac{\sin 2\theta}{2\omega} \qquad (4)$$

Thus, if the initiation of wing closure is controlled by time, this would occur when the time to the aperture T reaches the critical value T_0. This is the time-to-contact Tau strategy[1]. Closing the wings at a constant time T_0 prior to reaching the aperture is exactly equivalent to closing them at a constant distance D_0 ($=VT_0$) from the aperture, because the approach speed V_0 is constant for all flights, independent of aperture width (except for the narrowest aperture).

Thus, the proposed strategy for deciding whether to close the wings, and if so, to initiate the closure at a prescribed distance from the aperture, would involve two sequential computations: (i) From afar, a decision is made about whether or not to close the wings by determining whether the measured values of ω at various values of θ are larger or smaller their expected values, as specified by equation (1). If the former is true, then wing closure is required. (ii) If wing closure is deemed necessary, the distance to the aperture is monitored by tracking the angular velocity ω of the edge, and determining when ω in the direction of the edge θ exceeds the expected value for that direction for the prescribed value of D_0, as specified by equation (3).

This strategy would ensure that wing closure occurs only when the aperture is narrower than the wingspan, and that the closure, if it occurs, is initiated at a constant, prescribed distance from the aperture. In effect, the wing closure, if required, is triggered at the point where the black curve intersects the blue curve corresponding to the relevant aperture width in Fig. S3.

A schematic description of a neural circuit for performing these computations is proposed in Fig. S4. The upper part of Fig. S4 illustrates a circuit for determining whether an aperture that the bird is approaching is narrower than the bird's wingspan, W. As the bird approaches the aperture, the edge of the aperture successively stimulates angular velocity sensors 1,2, …n, which generate responses proportional to the angular velocities $\omega_1,\omega_2, \ldots \omega_n$. The angular velocity of the edge will depend upon the width of the aperture, and upon the viewing direction θ of the edge. Accordingly, the outputs of the angular velocity sensors are compared with the expected values for an aperture of width W for various viewing directions, as specified in equation (1). When any of these sensors registers an angular velocity that matches or exceeds the expected value, the output of the associated comparator will be set to 1, indicating that the aperture is narrower than the bird's wingspan W. Thus, switching of the output of any of the comparators from 0 to 1 indicates that wing closure will be necessary. In the presence of noise, the reliability of this decision can be improved by monitoring the outputs of several comparators. As the edge moves across the visual fields of successive angular velocity sensors, each comparator will switch to 1 if the aperture is narrower than the wingspan. Summation of the outputs of the comparators, and comparison with a pre-selected threshold (say, *n/2*, where n is the number of angular velocity sensors that have responded so far) will improve the reliability of the decision by ensuring that the aperture is deemed to be narrower than the wingspan only if at least half the number of comparators has switched to 1. The quality of this decision can be adjusted by varying the threshold. A threshold value lower than *n/2* will lead to detection that has greater sensitivity but is more prone to false alarms, whereas a value threshold value that is greater than *n/2* will have the opposite effect. The summation for determining whether wing closure is necessary can, of course, progress only until the bird has reached the critical distance D_0, at which point wing closure must be initiated if it is deemed necessary. Thus, the circuit can maintain a running total of n as well as the number of switched comparators until the critical distance D_0 is reached, to provide the most reliable decision about wing closure.

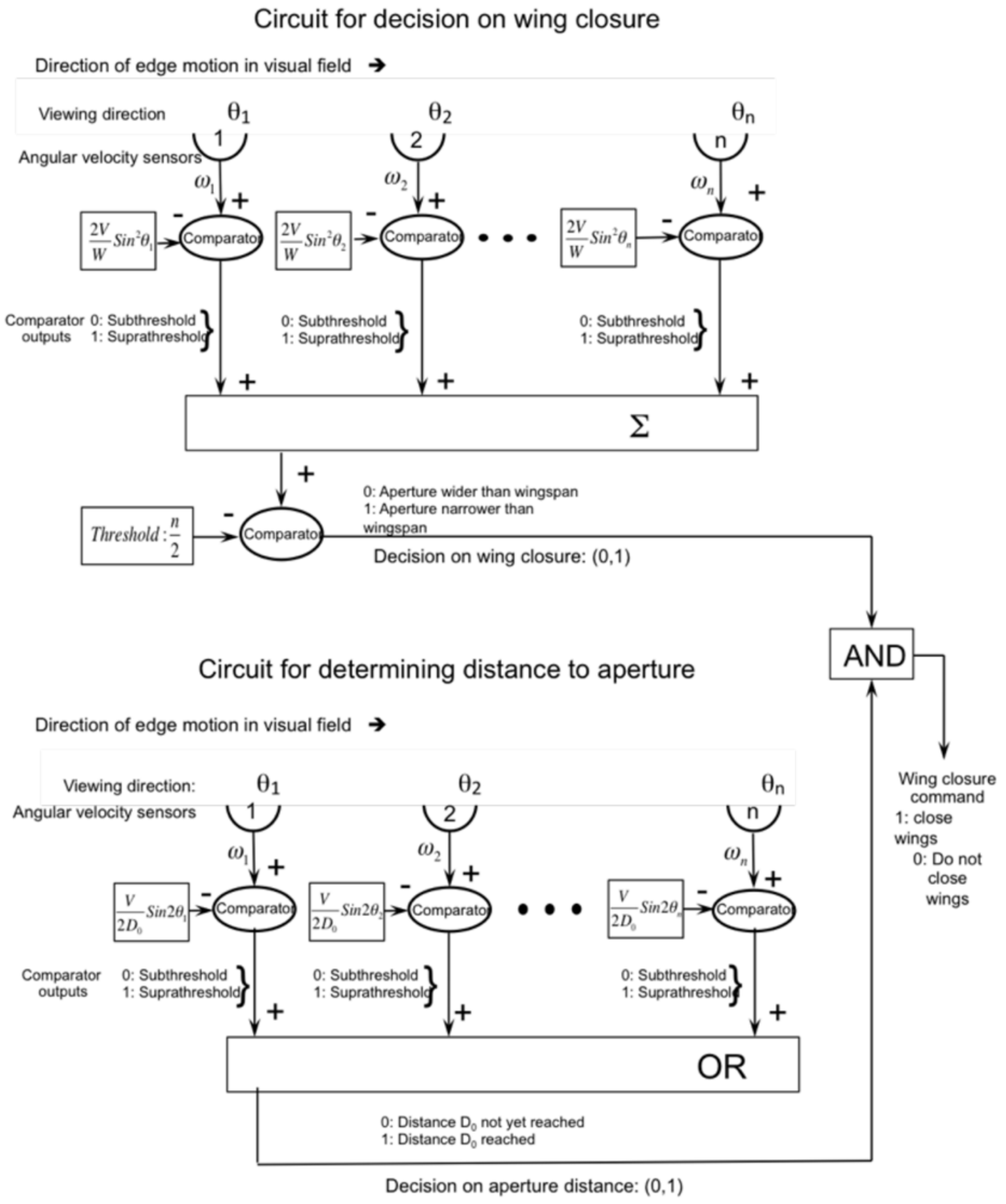

Supplementary Figure S4. Schematic description of neural circuits for (a) mediating a decision on wing closure, and (b) determining the distance to the aperture.

A circuit that ensures that wing closure, if necessary, is initiated at a prescribed distance D_0 from the aperture, is illustrated in the lower half of Fig. S4. This circuit receives its

inputs from the same set of angular velocity sensors. However, it compares the measured angular velocities against a different set of expected values, as prescribed in equation (3). The angular velocity of the edge when the bird is at a distance D_0 from the aperture will depend upon the width of the aperture, and upon the viewing direction θ of the edge. Accordingly, the outputs of the angular velocity sensors are compared with the expected values for an aperture distance D_0 for various viewing directions, as specified in equation (3). When any of these sensors registers an angular velocity that matches or exceeds the expected value, the output of the associated comparator will be set to 1, indicating that the aperture is at the critical distance D_0. The output of the inclusive OR operator at the final stage of the circuit will switch to 1 when any of the angular velocity sensors has exceeded its prescribed threshold, indicating that the distance to the aperture is now D_0.

The motor command to initiate wing closure is issued when two conditions are met: (a) the aperture is narrower than the wingspan, as determined by the upper circuit, and (b) the bird is at the critical distance D_0 from the aperture, as determined by the lower circuit. Accordingly, the motor command is derived by performing an 'AND' operation on the outputs of the two circuits.

Although the circuits described above have the flavour of electronic circuitry rather than neuronal circuitry, there is now evidence that computations such as the measurement of image angular velocity, and operations that involve thresholding, summation, and executing Boolean operations such as OR and AND can be performed by neural circuits[2,3].

We have seen above that constancy of the approach speed enables the calibration of distances and apertures directly in terms of optic flow magnitude, leading to a simple and robust scheme for controlling wing closure, as described above. In an earlier study, based on more limited information about the birds' behaviour when flying through narrow apertures[4], we had proposed that wing closure occurs when the angular velocity of the edge exceeds a threshold value. That model predicted that narrower apertures would elicit wing closure at larger distances from the aperture. In that study there was no information about birds' flight speeds, or about where they closed their wings in

relation to the aperture. We now have this information, and our present data shows that wing closure, if it occurs, takes place at a constant distance from the aperture, irrespective of the width of the aperture.

References

1 Lee, D. N. & Reddish, P. E. Plummeting gannets: A paradigm of ecological optics. *Nature* **293**, 293-294, doi:10.1038/293293a0 (1981).

2 Srinivasan, M. V., Poteser, M. & Kral, K. Motion detection in insect vision and navigation. *Vision Research* **39**, 2749-2766 (1999).

3 Squire, L. R. Fundamental Neuroscience. *Academic Press, Elsevier* (2013).

4 Schiffner, I., Vo, H. D., Bhagavatula, P. & Srinivasan, M. V. Minding the gap: in-flight body awareness in birds. *Frontiers in Zoology* **11** (2014).

RESULTS OF STATISTICAL ANALYSES

Legend

Gap: Aperture width; **Wing:** Wing closure/extension; **TD:** Time/Distance to aperture; **WCNWC:** Wing closure/non-closure

CVs of distance and time analysis:

Analysis of Variance of Aligned Rank Transformed Data

Table Type: Analysis of Deviance Table (Type III Wald F tests with Kenward-Roger df)
Model: Mixed Effects (lmer)
Response: art(CV)

		F	Df	Df.res	Pr(>F)
1	Gap	2.42623	6	120	0.0299874
2	Wing	1.60772	1	118	0.2073075
3	TD	0.15347	1	118	0.6959444
4	Gap:Wing	3.21777	6	118	0.0058008
5	Gap:TD	0.19345	6	118	0.9781261
6	Wing:TD	1.11731	1	118	0.2926564
7	Gap:Wing:TD	0.10198	6	118	0.9960473

Levene's Test for Homogeneity of Variance (center = median)

	Df	F value	Pr(>F)
group	27	0.937	0.5595
	124		

contrast	estimate	SE	df	t.ratio	p.value
G-1 - G-16	34.9599298	12.88407	120	2.713	0.1037
G-1 - G-3	35.5088737	13.4922	122	2.632	0.1256
G-1 - G-5	37.6385013	12.88407	120	2.921	0.0615
G-1 - G1	24.370992	16.15468	121	1.509	0.7392
G-1 - G3	36.2416513	14.97794	122	2.42	0.1994
G-1 - G5	46.9339382	13.19539	119	3.557	0.0095
G-16 - G-3	0.5489438	12.12347	119	0.045	1
G-16 - G-5	2.6785714	11.5499	118	0.232	1
G-16 - G1	-10.5889378	15.46411	122	-0.685	0.9932
G-16 - G3	1.2817215	13.92617	121	0.092	1
G-16 - G5	11.9740084	12.14364	119	0.986	0.9561
G-3 - G-5	2.1296276	12.12347	119	0.176	1
G-3 - G1	-11.1378817	15.7607	122	-0.707	0.992
G-3 - G3	0.7327776	14.24547	121	0.051	1
G-3 - G5	11.4250645	12.73582	121	0.897	0.9724
G-5 - G1	-13.2675093	15.46411	122	-0.858	0.9779
G-5 - G3	-1.39685	13.92617	121	-0.1	1
G-5 - G5	9.2954369	12.14364	119	0.765	0.9877
G1 - G3	11.8706593	16.71432	119	0.71	0.9918
G1 - G5	22.5629462	15.73222	121	1.434	0.7822
G3 - G5	10.6922869	14.22359	120	0.752	0.9889

Analysis of all flights:

Forward speed

Analysis of Variance of Aligned Rank Transformed Data

Table Type: Analysis of Deviance Table (Type III Wald F tests with Kenward-Roger df)
Model: Mixed Effects (lmer)
Response: art(FWDspd)

		F	Df	Df.res	Pr(>F)
1	Distance	4.6551	12	615	<.0001
2	Gap	48.7315	7	615	<.0001
3	Distance:Gap	1.0435	84	615	0.38144

Levene's Test for Homogeneity of Variance (center = median)

	Df	F value	Pr(>F)
group	103	0.782	0.9393
	621		

contrast	estimate	SE	df	t.ratio	p.value
G-1 - G-16	341.571429	24.23844	615	14.092	<.0001
G-1 - G-3	93.318681	24.23844	615	3.85	0.0033
G-1 - G-5	73.659341	24.23844	615	3.039	0.0504
G-1 - G0	-57.430582	24.50517	615	-2.344	0.2717
G-1 - G1	110.923077	24.23844	615	4.576	0.0002
G-1 - G3	86.318681	24.23844	615	3.561	0.0094
G-1 - G5	1.791209	24.23844	615	0.074	1
G-16 - G-3	-248.252747	24.23844	615	-10.242	<.0001
G-16 - G-5	-267.912088	24.23844	615	-11.053	<.0001
G-16 - G0	-399.002011	24.50517	615	-16.282	<.0001
G-16 - G1	-230.648352	24.23844	615	-9.516	<.0001
G-16 - G3	-255.252747	24.23844	615	-10.531	<.0001
G-16 - G5	-339.78022	24.23844	615	-14.018	<.0001
G-3 - G-5	-19.659341	24.23844	615	-0.811	0.9925
G-3 - G0	-150.749263	24.50517	615	-6.152	<.0001
G-3 - G1	17.604396	24.23844	615	0.726	0.9962
G-3 - G3	-7	24.23844	615	-0.289	1
G-3 - G5	-91.527473	24.23844	615	-3.776	0.0043
G-5 - G0	-131.089923	24.50517	615	-5.349	<.0001
G-5 - G1	37.263736	24.23844	615	1.537	0.7869
G-5 - G3	12.659341	24.23844	615	0.522	0.9995
G-5 - G5	-71.868132	24.23844	615	-2.965	0.0621
G0 - G1	168.353659	2.45E+01	615	6.87	<.0001
G0 - G3	143.749263	24.50517	615	5.866	<.0001
G0 - G5	59.221791	24.50517	615	2.417	0.235
G1 - G3	-24.604396	24.23844	615	-1.015	0.9721
G1 - G5	-109.131868	24.23844	615	-4.502	0.0002
G3 - G5	-84.527473	24.23844	615	-3.487	0.0122

3D speed

Analysis of Variance of Aligned Rank Transformed Data
Table Type: Analysis of Deviance Table (Type III Wald F tests with Kenward-Roger df)
Model: Mixed Effects (lmer)
Response: art(T3Dspd)

		F	Df	Df.res	Pr(>F)
1	Distance	20.04941	12	615	<.0001
2	Gap	19.20282	7	615	<.0001
3	Distance:Gap	0.42326	84	615	1

Levene's Test for Homogeneity of Variance (center = median)

	Df	F value	Pr(>F)
group	103	0.8751	0.7983
	621		

contrast	estimate	SE	df	t.ratio	p.value
G-1 - G-16	250.8021978	26.22553	615	9.563	<.0001
G-1 - G-3	146.8681319	26.22553	615	5.6	<.0001
G-1 - G-5	44.1428571	26.22553	615	1.683	0.6982
G-1 - G0	178.9699058	26.51414	615	6.75	<.0001
G-1 - G1	117.4065934	26.22553	615	4.477	0.0002
G-1 - G3	64.7472527	26.22553	615	2.469	0.2109
G-1 - G5	65.5934066	26.22553	615	2.501	0.1968
G-16 - G-3	-103.9340659	26.22553	615	-3.963	0.0021
G-16 - G-5	-206.6593407	26.22553	615	-7.88	<.0001
G-16 - G0	-71.832292	26.51414	615	-2.709	0.1217
G-16 - G1	-133.3956044	26.22553	615	-5.086	<.0001
G-16 - G3	-186.0549451	26.22553	615	-7.094	<.0001
G-16 - G5	-185.2087912	26.22553	615	-7.062	<.0001
G-3 - G-5	-102.7252747	26.22553	615	-3.917	0.0025
G-3 - G0	32.1017739	26.51414	615	1.211	0.9287
G-3 - G1	-29.4615385	26.22553	615	-1.123	0.9516
G-3 - G3	-82.1208791	26.22553	615	-3.131	0.0383
G-3 - G5	-81.2747253	26.22553	615	-3.099	0.0422
G-5 - G0	134.8270486	26.51414	615	5.085	<.0001
G-5 - G1	73.2637363	26.22553	615	2.794	0.0984
G-5 - G3	20.6043956	26.22553	615	0.786	0.9938
G-5 - G5	21.4505495	26.22553	615	0.818	0.9921
G0 - G1	-6.16E+01	26.51414	615	-2.322	0.2833
G0 - G3	-114.222653	26.51414	615	-4.308	0.0005
G0 - G5	-113.3764992	26.51414	615	-4.276	0.0006
G1 - G3	-52.6593407	26.22553	615	-2.008	0.4773
G1 - G5	-51.8131868	26.22553	615	-1.976	0.4993
G3 - G5	0.8461538	26.22553	615	0.032	1

Height

Analysis of Variance of Aligned Rank Transformed Data

Table Type: Analysis of Deviance Table (Type III Wald F tests with Kenward-Roger df)
Model: Mixed Effects (lmer)
Response: art(Height)

		F	Df	Df.res	Pr(>F)
1	Distance	0.22729	12	615	0.9971
2	Gap	9.750297	7	615	<.0001
3	Distance:Gap	0.069628	84	615	1

Levene's Test for Homogeneity of Variance (center = median)

	Df	F value	Pr(>F)
group	103	0.5404	0.9999
	621		

contrast	estimate	SE	df	t.ratio	p.value
G-1 - G-16	91.69230769	28.46231	615	3.222	0.0291
G-1 - G-3	-66.65934066	28.46231	615	-2.342	0.2726
G-1 - G-5	-16.94505495	28.46231	615	-0.595	0.9989
G-1 - G0	71.38644707	28.77552	615	2.481	0.2056
G-1 - G1	-96.10989011	28.46231	615	-3.377	0.0177
G-1 - G3	0.06593407	28.46231	615	0.002	1
G-1 - G5	19.75824176	28.46231	615	0.694	0.9971
G-16 - G-3	-158.3516484	28.46231	615	-5.564	<.0001
G-16 - G-5	-108.6373626	28.46231	615	-3.817	0.0037
G-16 - G0	-20.30586062	28.77552	615	-0.706	0.9968
G-16 - G1	-187.8021978	28.46231	615	-6.598	<.0001
G-16 - G3	-91.62637363	28.46231	615	-3.219	0.0293
G-16 - G5	-71.93406593	28.46231	615	-2.527	0.1859
G-3 - G-5	49.71428571	28.46231	615	1.747	0.6564
G-3 - G0	138.0457877	28.77552	615	4.797	0.0001
G-3 - G1	-29.45054945	28.46231	615	-1.035	0.969
G-3 - G3	66.72527473	28.46231	615	2.344	0.2713
G-3 - G5	86.41758242	28.46231	615	3.036	0.0508
G-5 - G0	88.33150202	28.77552	615	3.07	0.046
G-5 - G1	-79.16483516	28.46231	615	-2.781	0.1016
G-5 - G3	17.01098901	28.46231	615	0.598	0.9989
G-5 - G5	36.7032967	28.46231	615	1.29	0.9027
G0 - G1	-167.4963372	28.77552	615	-5.821	<.0001
G0 - G3	-71.32051301	28.77552	615	-2.479	0.2066
G0 - G5	-51.62820531	28.77552	615	-1.794	0.6243
G1 - G3	96.17582418	28.46231	615	3.379	0.0175
G1 - G5	115.8681319	28.46231	615	4.071	0.0014
G3 - G5	19.69230769	28.46231	615	0.692	0.9972

Analysis of wing closure flights:

Forward speed

Analysis of Variance of Aligned Rank Transformed Data

Table Type: Analysis of Deviance Table (Type III Wald F tests with Kenward-Roger df)
Model: Mixed Effects (lmer)
Response: art(FWDspd)

		F	Df	Df.res	Pr(>F)
1	Distance	2.7648	12	462	0.0012068
2	Gap	33.5791	6	462	<.0001
3	Distance:Gap	0.6999	72	462	0.9685635

Levene's Test for Homogeneity of Variance (center = median)

	Df	F value	Pr(>F)
group	90	1.2437	0.07958
	468		

contrast	estimate	SE	df	t.ratio	p.value
G-1 - G-16	229.34066	19.62852	462	11.684	<.0001
G-1 - G-3	25.82418	19.62852	462	1.316	0.8445
G-1 - G-5	29.38462	19.62852	462	1.497	0.7466
G-1 - G1	82.20588	26.19885	464	3.138	0.0298
G-1 - G3	53.09632	20.58502	462	2.579	0.1347
G-1 - G5	-10.80021	20.58502	462	-0.525	0.9985
G-16 - G-3	-203.51648	19.62852	462	-10.368	<.0001
G-16 - G-5	-199.95604	19.62852	462	-10.187	<.0001
G-16 - G1	-147.13478	26.19885	464	-5.616	<.0001
G-16 - G3	-176.24434	20.58502	462	-8.562	<.0001
G-16 - G5	-240.14087	20.58502	462	-11.666	<.0001
G-3 - G-5	3.56044	19.62852	462	0.181	1
G-3 - G1	56.3817	26.19885	464	2.152	0.3241
G-3 - G3	27.27215	20.58502	462	1.325	0.8401
G-3 - G5	-36.62439	20.58502	462	-1.779	0.5629
G-5 - G1	52.82126	26.19885	464	2.016	0.4058
G-5 - G3	23.71171	20.58502	462	1.152	0.9114
G-5 - G5	-40.18483	20.58502	462	-1.952	0.4469
G1 - G3	-29.10956	26.69478	463	-1.09	0.9308
G1 - G5	-93.00609	26.69478	463	-3.484	0.0097
G3 - G5	-63.89653	21.55757	463	-2.964	0.0496

3D speed

Analysis of Variance of Aligned Rank Transformed Data

Table Type: Analysis of Deviance Table (Type III Wald F tests with Kenward-Roger df)
Model: Mixed Effects (lmer)
Response: art(T3Dspd)

		F	Df	Df.res	Pr(>F)
1	Distance	8.801	12	462	<.0001
2	Gap	12.1192	6	462	<.0001
3	Distance:Gap	0.5795	72	4.62	0.99745

Levene's Test for Homogeneity of Variance (center = median)

	Df	F value	Pr(>F)
group	90	0.8549	0.8182
	468		

contrast	estimate	SE	df	t.ratio	p.value
G-1 - G-16	158.934066	20.58956	462	7.719	<.0001
G-1 - G-3	87.087912	20.58956	462	4.23	0.0006
G-1 - G-5	31.989011	20.58956	462	1.554	0.7119
G-1 - G1	20.841438	27.48402	463	0.758	0.9886
G-1 - G3	64.210377	21.59342	462	2.974	0.0483
G-1 - G5	58.52548	21.59342	462	2.71	0.0979
G-16 - G-3	-71.846154	20.58956	462	-3.489	0.0095
G-16 - G-5	-126.945055	20.58956	462	-6.166	<.0001
G-16 - G1	-138.092628	27.48402	463	-5.024	<.0001
G-16 - G3	-94.723689	21.59342	462	-4.387	0.0003
G-16 - G5	-100.408586	21.59342	462	-4.65	0.0001
G-3 - G-5	-55.098901	20.58956	462	-2.676	0.1067
G-3 - G1	-66.246474	27.48402	463	-2.41	0.1965
G-3 - G3	-22.877535	21.59342	462	-1.059	0.9394
G-3 - G5	-28.562432	21.59342	462	-1.323	0.8411
G-5 - G1	-11.147573	27.48402	463	-0.406	0.9997
G-5 - G3	32.221366	21.59342	462	1.492	0.7495
G-5 - G5	26.536469	21.59342	462	1.229	0.8827
G1 - G3	43.368939	28.00384	463	1.549	0.715
G1 - G5	37.684042	28.00384	463	1.346	0.8299
G3 - G5	-5.684897	22.61431	463	-0.251	1

Height

Analysis of Variance of Aligned Rank Transformed Data

Table Type: Analysis of Deviance Table (Type III Wald F tests with Kenward-Roger df)
Model: Mixed Effects (lmer)
Response: art(Height)

		F	Df	Df.res	Pr(>F)
1	Distance	0.26841	12	462	0.99356
2	Gap	10.04928	6	462	<.0001
3	Distance:Gap	0.056114	72	462	1

Levene's Test for Homogeneity of Variance (center = median)

	Df	F value	Pr(>F)
group	90	0.8378	0.8479
	468		

contrast	estimate	SE	df	t.ratio	p.value
G-1 - G-16	120.5494506	21.6398	462	5.571	<.0001
G-1 - G-3	10.12087912	21.6398	462	0.468	0.9992
G-1 - G-5	6.45054945	21.6398	462	0.298	0.9999
G-1 - G1	-70.05122005	28.88573	463	-2.425	0.1904
G-1 - G3	6.38301627	22.69483	462	0.281	1
G-1 - G5	30.25294418	22.69483	462	1.333	0.8361
G-16 - G-3	-110.4285714	21.6398	462	-5.103	<.0001
G-16 - G-5	-114.0989011	21.6398	462	-5.273	<.0001
G-16 - G1	-190.6006706	28.88573	463	-6.598	<.0001
G-16 - G3	-114.1664343	22.69483	462	-5.031	<.0001
G-16 - G5	-90.29650637	22.69483	462	-3.979	0.0016
G-3 - G-5	-3.67032967	21.6398	462	-0.17	1
G-3 - G1	-80.17209917	28.88573	463	-2.775	0.0829
G-3 - G3	-3.73786285	22.69483	462	-0.165	1
G-3 - G5	20.13206506	22.69483	462	0.887	0.9744
G-5 - G1	-76.5017695	28.88573	463	-2.648	0.1142
G-5 - G3	-0.06753318	22.69483	462	-0.003	1
G-5 - G5	23.80239473	22.69483	462	1.049	0.9422
G1 - G3	76.43423632	29.4321	463	2.597	0.1292
G1 - G5	100.3041642	29.4321	463	3.408	0.0125
G3 - G5	23.86992791	23.76772	463	1.004	0.9529

Analysis of wing closure versus non-wing closure flights:

Forward speed

Analysis of Variance of Aligned Rank Transformed Data

Table Type: Analysis of Deviance Table (Type III Wald F tests with Kenward-Roger df)
Model: Mixed Effects (lmer)
Response: art(FWDspd)

		F	Df	Df.res	Pr(>F)
1	WCNWC	0.29914	1	48	0.58695
2	Gap	0.26658	5	45	<.0001
3	WCNWC:Gap	0.40986	5	46	0.83949

Levene's Test for Homogeneity of Variance (center = median)

	Df	F value	Pr(>F)
group	11	0.4597	0.9191
	50		

3D speed

Analysis of Variance of Aligned Rank Transformed Data

Table Type: Analysis of Deviance Table (Type III Wald F tests with Kenward-Roger df)
Model: Mixed Effects (lmer)
Response: art(T3Dspd)

		F	Df	Df.res	Pr(>F)
1	WCNWC	0.43003	1	46	0.51518
2	Gap	0.6517	5	45	0.66167
3	WCNWC:Gap	1.23997	5	45	0.30626

Levene's Test for Homogeneity of Variance (center = median)

	Df	F value	Pr(>F)
group	11	1.0677	0.4053
	50		

Height

Table Type: Analysis of Deviance Table (Type III Wald F tests with Kenward-Roger df)
Model: Mixed Effects (lmer)
Response: art(Height)

		F	Df	Df.res	Pr(>F)
1	WCNWC	7.94404	1	45	0.0070965
2	Gap	0.74305	5	44	0.5954019
3	WCNWC:Gap	0.94696	5	44	0.4602417

Levene's Test for Homogeneity of Variance (center = median)

	Df	F value	Pr(>F)
group	11	0.1963	0.9971
	50		

contrast	estimate	SE	df	t.ratio	p.value
NWC - WC	-13.05764	4.632806	45	-2.819	0.0071
